# Assessing Pediatric Feeding Disorders by Domain in Complex Aerodigestive Patients

**DOI:** 10.7759/cureus.17409

**Published:** 2021-08-24

**Authors:** Erin Alexander, Andrea Armellino, Julie Buchholtz, Laura Dinnes, Molissa Hager, Beth Ruechel, Dana B Steien, R. Paul Boesch, Shelagh Cofer, Rayna Grothe

**Affiliations:** 1 Pediatric Gastroenterology, Mayo Clinic, Rochester, USA; 2 Division of Endocrinology and Diabetes, Mayo Clinic, Rochester, USA; 3 Department of Pharmacy, Mayo Clinic, Rochester, USA; 4 Division of Nursing, Mayo Clinic, Rochester, USA; 5 Pediatric Pulmonology, Mayo Clinic, Rochester, USA; 6 Pediatric Otorhinolaryngology, Mayo Clinic, Rochester, USA

**Keywords:** nutrition, feeding skill, aspiration, growth, aerodigestive, pediatrics

## Abstract

Objective: Pediatric feeding disorder (PFD) is defined as impaired oral intake, associated with dysfunction in at least one of four domains: medical, nutritional, feeding skill, and/or psychosocial. The pediatric aerodigestive patient presents with conditions impacting airway, breathing, feeding, swallowing, or growth. The objective of the study was to determine the prevalence of PFD and dysfunctional domain, in the aerodigestive patient presenting to a tertiary aerodigestive clinic.

Methods: Twenty-five charts from patients enrolled in Mayo Clinic Children’s Center Aerodigestive Program were retrospectively reviewed for documentation of dysfunction within the four feeding disorder domains. Results from the aerodigestive triple scope, functional endoscopic evaluation of swallow (FEES), and videofluoroscopic swallow study (VFSS) were recorded. Height and weight z-scores were compared between the initial assessment and 6-12 months later.

Results: Median age was 20 months (range 2-81 months). Of the patients, 100% (n = 25) had dysfunction in at least one PFD domain. The domain identified most frequently was medical dysfunction (96%; n = 24). Feeding dysfunction was observed in 76% (n = 19). Psychosocial dysfunction was observed in 76% (n = 19). Nutritional dysfunction was observed in 60% (n = 15). Dysfunction in three or greater domains was seen in 80% (n = 20). Weight z-score increased in 76% (n = 19) of patients 6 to 12 months after the initial aerodigestive evaluation.

Conclusion: Aerodigestive patients frequently have PFD and utilizing the consensus definition of PFD at intake may enhance clinical assessment and therapeutic evaluation, and provide a framework to measure outcomes in this heterogeneous patient population.

## Introduction

Pediatric feeding disorders (PFD) affect up to 5% of infants and toddlers [1], and up to 70% of children with chronic medical conditions [2]. Feeding disorders can manifest with many different signs and symptoms. Some cases are easily identified, while others are more challenging, particularly in the presence of normal growth. Unrecognized PFDs can result in severe consequences including compromised immune system, chronic aspiration, growth failure, and death [3]. Therefore, early identification and intervention are critical.

Previous descriptions of PFD did not include the functional limitations that these patients experience [4]. PFD had previously been described as organic versus non-organic or focused on a single system or diagnosis [1]. Burklow et al. evaluated 103 children presenting to a feeding clinic and documented the high prevalence of behavioral issues (85%) as well as the presence of structural abnormalities (57%), cardiorespiratory problems (7%), and neurological conditions (73%). This suggests that feeding disorders have both physiological and behavioral problems interacting to result in PFD [5]. An expert group in 2019 published a consensus definition of PFD, which recognized functional impairment in the diagnosis of PFD, based upon the World Health Organization International Classification of functioning, disability, and health [4]. The new consensus definition of PFD is defined as the impaired oral intake that is not age-appropriate, and is associated with at least one dysfunctional domain: medical, nutritional, feeding skill, and psychosocial [4]. The new definition allows for a unified and consistent approach to identifying patients affected by PFD.

A consensus statement defined pediatric aerodigestive disease as multiple and interrelated congenital and/or acquired conditions affecting airway, breathing, feeding, swallowing, or growth that requires a coordinated interdisciplinary diagnostic and therapeutic approach to achieve optimal outcomes [4]. Essential components of an aerodigestive program include clinical swallowing evaluations: fiberoptic endoscopic evaluations of swallow (FEES), videofluoroscopic swallow studies (VFSS), and direct feeding therapy [6]. Aerodigestive clinics provide an opportunity to appropriately diagnose and treat patients with PFD. Thus, we used the consensus definition of PFDs in our tertiary pediatric aerodigestive program, to examine the prevalence of PFD, as well as each domain subtype within this population. This article was previously presented as a meeting abstract at the 2019 North American Society for Pediatric Gastroenterology, Hepatology and Nutrition (NASPGHAN) annual meeting on October 18, 2020.

## Materials and methods

**Figure 1:**
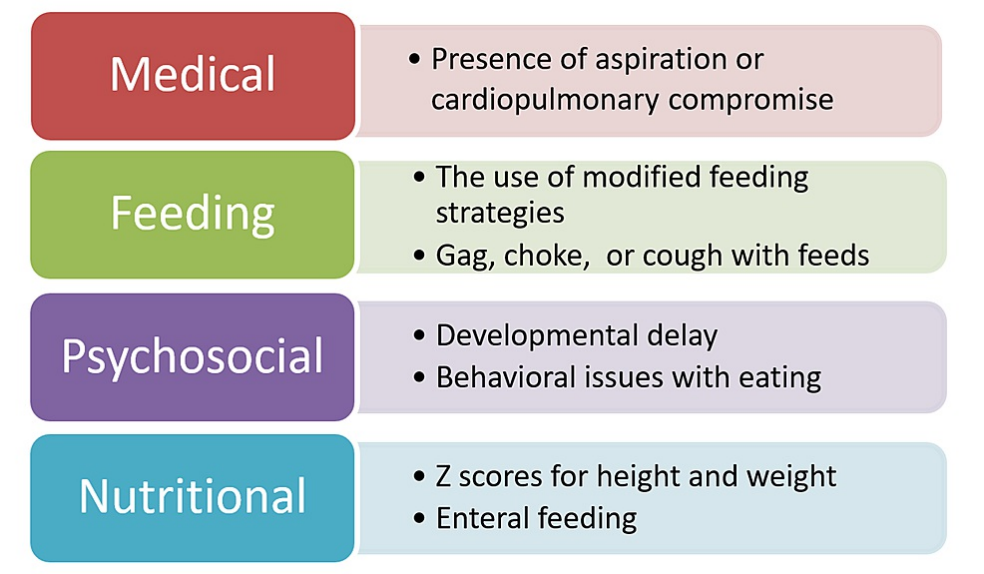
Domains of dysfunction in pediatric feeding disorders.

This was a retrospective study approved by the Mayo Clinic Institutional Review Board. Twenty-five patients, evaluated before September 2018, were sequentially chosen from the Mayo Clinic Children’s Center Aerodigestive Program database. Date of the initial visit allowed for a 6-12 month follow-up. Enrollment in the aerodigestive program requires phone intake/screening. These children must meet two major criteria or one major and two minor criteria to be enrolled in the interdisciplinary program. Criteria are listed in Appendix 1. Each electronic medical record was reviewed for evidence of PFD based upon the consensus statement. Inadequate oral intake was documented, and identification of dysfunction in specific domains (medical, nutritional, feeding skill level, and psychosocial) was documented. See Figure 1.

Definitions of the domains of PFD were based on the paper by Goday et al. in 2019 [4]. The medical dysfunction domain is defined as the presence of cardiopulmonary compromise or aspiration. The cardiopulmonary compromise was defined as having supplemental oxygen, recurrent pneumonia, or tracheostomy dependence. The presence or absence of aspiration was reported by parents during the initial intake. Patients met criteria for the nutritional domain if weight z-scores indicated malnutrition (< −2.0), and/or if a patient was using an enteral feeding tube [7]. Dysfunctional feeding skills were noted if modified feeding strategies were required (e.g. thickened feeds or specialized feeding equipment), or if patients had gagging or coughing with feeds. The psychosocial dysfunction domain was diagnosed in patients with avoidant feeding behaviors, inappropriate caregiver feeding management, disruption of social functioning while feeding, or disruption of the caregiver-child relationship during feeding [4].

All patients had undergone a comprehensive aerodigestive evaluation, which consisted of esophagogastroduodenoscopy, pH impedance probe, microlaryngoscopy, bronchoscopy, FEES, and VFSS. Abnormal airway was defined as the presence of clefts (laryngeal cleft or submucosal palatal cleft), stenosis, tracheomalacia, or post-surgical anatomic abnormalities (most commonly from a tracheostomy or subglottic stenosis repair). Bronchitis was defined as >17% polymorphoneutrophils (PMNS) on bronchoalveolar lavage (BAL) [8-11]. Height and weight z-scores were recorded at the time of the initial visit and 6-12 months later.

For statistical analysis, JMP software (SAS Institute, Cary, North Carolina) was used. A matched pair t-test was used to compare the z-scores for height and weight over time. Chi-square goodness-of-fit test was used to analyze for the association of (1) aspiration on VFSS and presence of bronchitis on BAL, (2) penetration on VFSS and bronchitis on BAL, and (3) aspiration on VFSS and feeding skill dysfunction (gagging, choking, and coughing with feeds). Gagging, choking, and coughing with feeds were compared both independently and together. In addition, the association between laryngeal penetration on VFSS and symptoms of feeding skill dysfunction was analyzed. Association of gagging, coughing, and choking and the presence of bronchitis was also evaluated. The association of either aspiration or penetration on VFSS and FEES, and the presence of bronchitis, was assessed. FEES and VFSS results were documented individually. The potential difference in results between FEES and VFSS was assessed with a Fisher exact test. To assess if the patients who had an intervention for PFD were more likely to have weight gain, a Wilcoxon rank-sum test was used.

## Results

**Figure 2:**
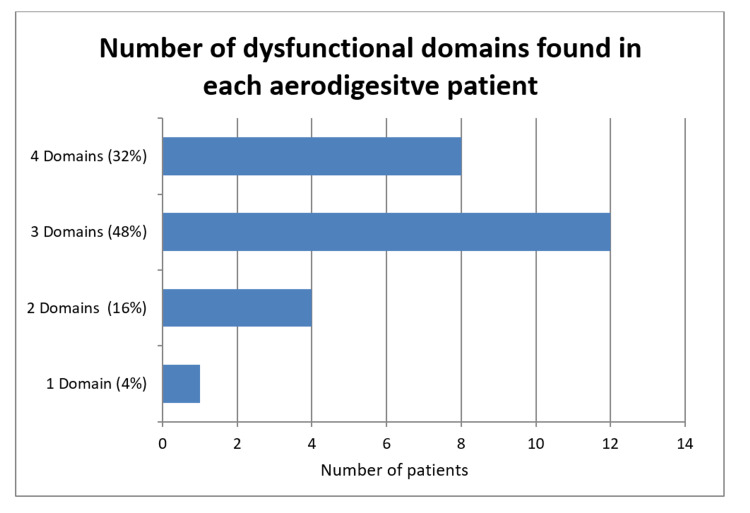
Number of dysfunctional domains found in each patient. Specific domains impacted in each individual patient are shown in Table 1.

**Figure 3:**
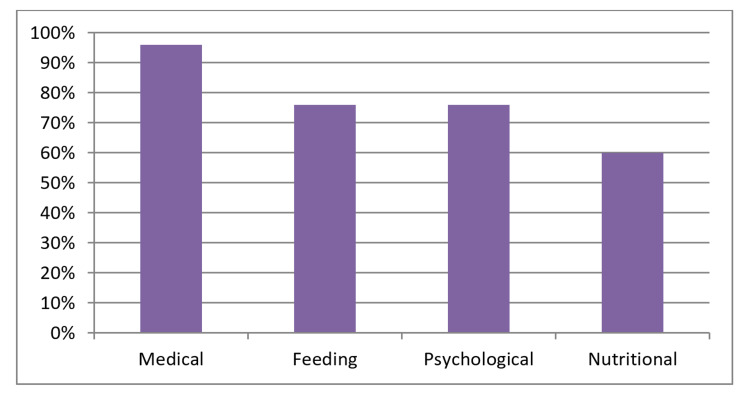
Frequency of domain (% of 25 patients).

Twenty-five patients were evaluated. Of the patients, 56% (n = 14) were female and ages ranged from 2 to 81 months (median 20 months). Patient characteristics are listed in Table 1. All patients met the criteria of impaired oral intake inappropriate for age and met diagnostic criteria for dysfunction in at least one PFD domain. Dysfunction in all four PFD domains was observed in 32% of the patients (n = 8). Dysfunction in three domains was observed in 48% (n = 12). Dysfunction in two domains was observed in 16% (n = 4). Dysfunction in one domain was observed in 4% (n = 1) and was noted to be feeding dysfunction (Figure 2). The PFD domain with the highest incidence was medical dysfunction, at 96% of the patients (n = 24). Feeding dysfunction was observed in 76% (n = 19). Psychosocial dysfunction was observed in 76% (n = 19). Nutritional dysfunction was observed in 60% (n = 15) (Figure 3).

**Table 1:** Patient demographics, diagnoses, and specific domain(s) of dysfunction. CHARGE, coloboma, heart defect, retarded growth and development, genital abnormalities, and ear abnormalities.

Patient	Age (months)	Sex	Diagnosis	Disturbance of oral intake inappropriate for age	Medical dysfunction	Feeding skill dysfunction	Psychosocial dysfunction	Nutritional dysfunction
1	21	Male	Laryngomalacia, hypotonia, tracheostomy dependence	Yes	Yes	Yes	Yes	Yes
2	14	Male	Congenital heart disease, subglottic stenosis, recurrent pneumonia	Yes	Yes	Yes	Yes	No
3	11	Female	Bronchopulmonary dysplasia, patent ductus arteriosus	Yes	Yes	Yes	Yes	Yes
4	32	Female	Laryngomalacia, dysphagia, aspiration	Yes	Yes	Yes	No	Yes
5	17	Female	Laryngomalacia, dysphagia, reflux	Yes	Yes	Yes	No	No
6	73	Male	CHARGE syndrome	Yes	Yes	No	Yes	Yes
7	2	Female	Myelomeningocele, hydrocephalus	Yes	Yes	No	Yes	Yes
8	4	Female	Laryngomalacia, reflux	Yes	No	Yes	No	No
9	11	Male	Trisomy 21, ventriculoseptal defect, Hirschsprung’s disease	Yes	Yes	No	Yes	Yes
10	27	Female	Trisomy 21, chronic pneumonia, atrial septal defect	Yes	Yes	Yes	Yes	No
11	12	Male	Chromosomal abnormality, sinusitis, reflux, hypotonia	Yes	Yes	Yes	Yes	Yes
12	83	Female	Trisomy 21, tracheoesophageal fistula	Yes	Yes	Yes	Yes	Yes
13	25	Male	Recurrent infections	Yes	Yes	Yes	No	No
14	5	Female	Cough and dysphagia	Yes	Yes	Yes	No	No
15	23	Male	Recurrent infections	Yes	Yes	Yes	Yes	Yes
16	8	Female	Trisomy 21, laryngomalacia	Yes	Yes	Yes	Yes	Yes
17	84	Female	Recurrent infections, dysphagia	Yes	Yes	No	Yes	Yes
18	7	Female	Pierre-Robin sequence, cleft palate	Yes	Yes	Yes	Yes	Yes
19	20	Male	Tracheoesophageal fistula, esophageal stricture	Yes	Yes	Yes	Yes	No
20	14	Male	Chromosomal abnormality	Yes	Yes	Yes	Yes	No
21	35	Female	Stickler syndrome, reflux	Yes	Yes	No	Yes	Yes
22	15	Female	Tracheostomy dependence	Yes	Yes	Yes	Yes	Yes
23	34	Male	Chromosomal abnormality, aspiration	Yes	Yes	No	Yes	Yes
24	13	Female	Trisomy 21, subglottic stenosis	Yes	Yes	Yes	Yes	No
25	59	Male	Dysphagia, recurrent infections, oxygen dependence	Yes	Yes	Yes	No	No

The aerodigestive workup revealed 84% of the patients (n = 21) had an airway abnormality (specific diagnoses are listed in Table 2). Of the patients, 72% (n = 18) had bronchitis, and 12% (n = 3) had esophagitis. Aspiration and/or penetration on FEES and VFSS was observed in 40% of patients (n = 10). There was no association between esophagitis and aspiration/penetration, but there was an association of bronchitis with aspiration/penetration (p = 0.0054). Seventeen patients successfully completed both a VFSS and swallow evaluation with FEES. Three patients had FEES, but no VFSS, and two patients had a VFSS without FEES. Of the seventeen patients that had both FEES and VFSS, four patients had abnormal VFSS and normal FEES. Zero patients had abnormal FEES and normal VFSS.

**Table 2:** List of airway abnormalities diagnosed on aerodigestive evaluation.

List of documented airway abnormalities
Laryngeal cleft
Subglottic web
S/p supraglottoplasty
Lymphoid/tonsillar/adenoid hyperplasia
Pharyngeal narrowing
Tracheomalacia/bronchomalacia/pharyngomalacia
Tall arytenoids
Deep laryngeal notch
Supraglottic obstruction
Subglottic stenosis
Cleft palate
Mucosal cyst
Tracheostomy

**Figure 4:**
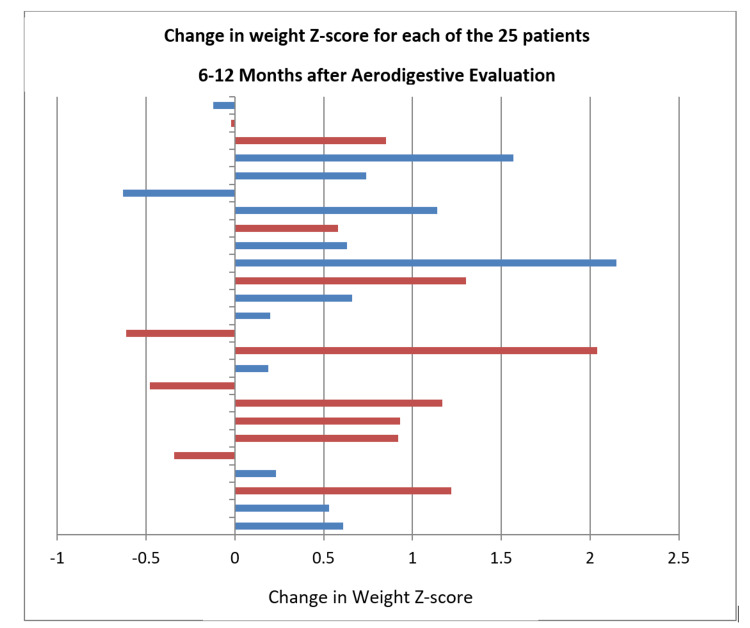
Change in weight-for-age z-score 6-12 months after aerodigestive evaluation. Patients given a documented change in feeding recommendation are red, and patients *without *a recommended change to their feeding regimen are blue.

At the conclusion of the aerodigestive evaluation, 64% (n = 16) had documented disordered feeding mentioned on the problem list and 48% (n = 12) had documented recommendations to alter the feeding method. Improved z-score for weights was seen in 76% (n = 19) of patients 6 to 12 months post-aerodigestive evaluation. The mean change in z-score for weight for this cohort was +0.62 with a median of +0.63 (Figure 4). Children who received recommendations to alter their feeding method had a mean z-score increase of +0.63, and a median weight z-score increase of +0.885; while those who did not receive recommendations on feeding changes had a mean weight z-score increase of +0.61 and a median weight z-score increase of +0.61. Despite the trend toward greater weight gain in the group who received feeding recommendations, this was not statistically significant (p = 0.6635).

## Discussion

Dysfunction in the four PFD domains, medical, nutritional, feeding skills, and psychosocial/behavioral, should be addressed in order to effectively treat PFDs. Within the framework of the consensus definition of PFD, this cohort of pediatric aerodigestive patients demonstrated evidence of a feeding disorder, with 95% of patients being involved in at least three domains. This finding demonstrates the pervasiveness of PFD and the complexity of this disorder in aerodigestive patients.

The consensus definition of PFD provides a global assessment of all the functional factors that will result in a pediatric feeding disorder in the aerodigestive patient. Impairment in one domain may adversely impact function in another domain. For example, severe dysphagia (feeding skill dysfunction) associated with aspiration (medical dysfunction) secondary to a congenital laryngeal cleft may result in feeding aversion (psychosocial dysfunction) and subsequent weight loss (nutritional dysfunction). Repairing the laryngeal cleft may improve aspiration. However, specific measures to address feeding aversion and malnutrition will need to be implemented for the child to fully recover. The findings in our retrospective review of 25 aerodigestive patients also demonstrate the connection between dysfunctional domains. We found that bronchitis was associated with aspiration or penetration. Aspiration, dysfunction in a medical domain, was noted on VFSS or FEES in 40% of patients. The aspiration was secondary to swallowing dysfunction, a feeding skill dysfunction. As all children in this review had difficulties with oral intake, looking also at the impact of psychosocial dysfunction and nutritional dysfunction will provide a more complete picture of the child’s feeding disorder.

The VFSS is the gold standard for assessing for aspiration during swallow. Our study did not show a statistically significant correlation between symptoms of feeding skill dysfunction (gagging, choking, and coughing with feeds) and the presence of aspiration/penetration on FEES/VFSS. This is similar to a previous study, where no single symptom in children under two years old, correlated with aspiration on VFSS [12]. Symptoms included gastrointestinal (GI) symptoms (choking, gagging, reflux, vomiting, poor feeding, and slow feeding) and pulmonary symptoms (coughing, noisy breathing, congestion, spells of respiratory distress, recurrent pneumonia, and supplemental oxygen requirements).

However, the Pediatric Eating Assessment Tool-10 (pEAT-10), a 10-question screening tool (questionnaire can be found in Appendix 2) has been shown to predict aspiration in children with esophageal atresia and children with neurological impairments [13,14]. The pEAT-10 questionnaire asks about symptoms of coughing and choking while eating but also asks more specific functional questions within the psychosocial/behavioral domain, the nutritional domain, and the feeding skill domain [13,14]. For example: Does the swallowing problem of my child interfere with the ability to go out for meals (psychosocial domain)? Does my child not gain weight due to his/ her swallowing problem (nutrition domain)? Does swallowing liquids or solids take extra effort for my child (feeding skill domain)? Thus, a questionnaire devised to evaluate for dysfunction in feeding domains may lead to a more accurate assessment of the aerodigestive patients underlying pathophysiology, facilitating more effective treatment.

A recently published consensus statement regarding the structure and function of pediatric aerodigestive disorders identified 20 research priorities for this population [6]. Of these, many were directly or indirectly related to PFD including quality of life, oral feeding status, and gastrostomy tube removal, swallowing indices by VFSS and FEES, family satisfaction, growth indices, aspiration biomarkers, and control of aspiration [6]. Currently, there is minimal outcome data regarding the pediatric multidisciplinary aerodigestive evaluation. A single-institution retrospective study published in 2019 showed decreased length of time to diagnosis and fewer radiologic and anesthetic exposures in patients who were evaluated in an aerodigestive program when compared to patients with similar conditions evaluated prior to the development of the program [15]. Another retrospective study showed a decrease in hospital days and lower costs associated with a multidisciplinary aerodigestive evaluation when compared to state averages [16]. Our retrospective review showed a significant increase in weight z-scores 6-12 months after initial evaluation with a trend towards greater weight gain in those patients who had specific feeding interventions. Evaluating for PFD utilizing the standardized approach to document dysfunction within the four important domains will provide the opportunity for analysis of PFD in the aerodigestive patient and outcomes measurement across institutions.

In this study, 100% of the aerodigestive patients reviewed had a PFD, with most having dysfunction noted in multiple domains. Addressing and treating these PFDs could improve growth and quality of life for these patients [17]. Improved z-score for weights was seen in 76% of patients 6 to 12 months post aerodigestive evaluation with the median change in z-score for weight +0.63 (p = 0.0004). The subset of patients within the improved z-score for weight group that had documented recommendations to alter the feeding method had a greater median change in z-score for the weight of +0.885. The overall improvement in z-score for weight in these patients 6 to 12 months post evaluation shows that addressing and treating PFDs in the aerodigestive patient has the potential to improve growth.

Untreated PFD can lead to failure to thrive and subsequent negative sequelae to cognitive and physical health [18]. Medically complex children have been shown to have slower progress than their non-medically complex counterparts during feeding therapy [19], which highlights the importance of early intervention. A multidisciplinary aerodigestive evaluation is a key opportunity to diagnose and manage PFD, as other areas of care are being coordinated simultaneously.

This study is limited by its small sample size and retrospective nature. While all 25 of these aerodigestive patients met pediatric feeding disorder criteria found in the 2019 consensus statement, it would be helpful to evaluate more patients across institutions to make it more generalizable. While the aerodigestive patient population is heterogeneous, each of the patients in our cohort met the same screening criteria (see Appendix 1). Thus, we feel it would be likely that results will be similar in another randomly selected group of patients from our Aerodigestive Clinic database. New research with prospective studies is suggested. Future studies should assess the long-term outcomes in the improvement of growth and nutrition, with the possibility of a cure for PFD.

## Conclusions

In conclusion, in this study cohort of 25 aerodigestive patients, at least one dysfunctional domain for PFD was met by all 25 patients with most patients having dysfunction in three or greater domains. The most commonly identified PFD domain in this population was medical dysfunction. Aerodigestive patients have a high incidence of PFD, and utilizing the consensus definition of PFD at intake may enhance clinical evaluation and interventions to improve outcomes. The utilization of a PFD tool that captures dysfunction in all four feeding disorder domains may help standardize aerodigestive care across institutions and allow for the evaluation of outcome data. Based on the pervasiveness of feeding disorders in aerodigestive patients, this should be a standard in the interdisciplinary aerodigestive evaluation.

## Appendices

Appendix 1

**Table 3:** Criteria for referral to Mayo Clinic Children’s Center Interdisciplinary Aerodigestive Clinic. Criteria for referral to Mayo Clinic Children’s Center Interdisciplinary Aerodigestive Clinic are assessed at phone intake. Two major or one major and two minor criteria are required for formal aerodigestive evaluation. *Subglottic stenosis, glottic stenosis, laryngeal web, laryngeal atresia, tracheal stenosis, and complete tracheal rings. **Trisomy 21; coloboma, heart defect, retarded growth and development, genital abnormalities, and ear abnormalities (CHARGE) syndrome; 22q11; vertebral, anal, tracheoesophageal, radial and renal anomalies (VATER)/vertebral defects, anal atresia, cardiac defects, tracheoesophageal fistula, renal anomalies, and limb abnormalities (VACTERL); Pfieffer syndrome; Opitz syndrome; craniofacial syndromes; Cornelia deLange syndrome; and Cri du Chat syndrome. GERD - gastroesophageal reflux disease.

Major conditions	Minor conditions
Airway stenosis*	Developmental delay
Aspiration (known or suspected)	Feeding problems
Chronic lung disease	GERD
Global CNS impairment	Laryngomalacia
Chiari malformation	Noisy breathing
Esophageal dysmotility	Recurrent chest infections
Esophageal stricture	Tracheomalacia/bronchomalacia
Genetic condition (listed)**	
Laryngeal cleft (current or prior)	
Sleep-disordered breathing	
Tracheoesophageal fistula (current or prior)	
Tracheostomy	
Vocal cord paralysis	

Appendix 2

Pediatric Eating Assessment Tool-10 (pEAT-10) [14].

pEAT-10: Each scored from 0 (no problem) to 4 (severe problem).

1. My child does not gain weight due to his/her swallowing problem.

2. Swallowing problem of my child interferes with our ability to go out for meals.

3. Swallowing liquids takes extra effort for my child.

4. Swallowing solids takes extra effort for my child.

5. My child gags during swallowing.

6. My child acts like he/she is in pain while swallowing.

7. My child does not want to eat.

8. Food sticks in my child’s throat and my child chokes while eating.

9. My child coughs while eating.

10. Swallowing is stressful for my child.
